# Three-Year-Olds’ Understanding of the Consequences of Joint Commitments

**DOI:** 10.1371/journal.pone.0073039

**Published:** 2013-09-04

**Authors:** Maria Gräfenhain, Malinda Carpenter, Michael Tomasello

**Affiliations:** 1 Department for Child and Adolescent Psychiatry, University of Leipzig, Leipzig, Germany; 2 Department of Developmental Psychology, University of Göttingen, Göttingen, Germany; 3 Department of Developmental and Comparative Psychology, Max Planck Institute for Evolutionary Anthropology, Leipzig, Germany; Centre for Coevolution of Biology & Culture, University of Durham, United Kingdom

## Abstract

Here we investigate the extent of children’s understanding of the joint commitments inherent in joint activities. Three-year-old children either made a joint commitment to assemble a puzzle with a puppet partner, or else the child and puppet each assembled their own puzzle. Afterwards, children who had made the joint commitment were more likely to stop and wait for their partner on their way to fetch something, more likely to spontaneously help their partner when needed, and more likely to take over their partner’s role when necessary. There was no clear difference in children’s tendency to tattle on their partner’s cheating behavior or their tendency to distribute rewards equally at the end. It thus appears that by 3 years of age making a joint commitment to act together with others is beginning to engender in children a “we”-intentionality which holds across at least most of the process of the joint activity until the shared goal is achieved, and which withstands at least some of the perturbations to the joint activity children experience.

## Introduction

Humans act together in various ways every day, achieving goals one individual alone could never achieve. Recently, research in developmental psychology has begun to address the question of when young children begin to act together with others intentionally in pursuit of joint goals, and what they understand about various aspects of joint activities [1]. There is increasing evidence that children begin to participate in joint activities early in ontogeny, suggesting that they are both motivated to act together with others and able to coordinate their actions with others. Already in their second year of life, infants engage in collaborative activities with adults such as in ritualized or even novel social games and simple problem-solving tasks [2]–[6]. Somewhat later, at the beginning of their third year of life, they also begin to act jointly with same-aged peers [7]–[13]. Moreover, a recent study suggests that young children act together with their partner with an understanding of the other as a mental agent with whom they share intentional states [14]. Together, these findings suggest that young children may have an understanding of joint activities that goes beyond just coordinating actions and includes shared goals and intentions [15]–[22].

However, one of the challenges when studying young children’s understanding of joint activity is to distinguish whether children really understand themselves as an active partner in a joint activity or not, since when all goes well it is often not so easy to know how jointly partners are acting, even in adults. If two people are walking side by side, an observer might not know whether they were doing so together or not until something happens. That is, if one stops to tie his shoes and the other keeps going, it is likely that they are not walking together in any meaningful way. In contrast, if one stops and the other feels obligated to wait and to help if needed, an observer would know that they were walking together. Their behavior would indicate that they are jointly committed to act together, with all the rights and obligations that this entails [23]–[26].

Previous studies have shown that young children have some basic understanding of some simple joint commitments. For example, children under 2 years of age will wait for their partners when needed and help by attempting to reengage them during interruptions in their joint activity [3], [4], [14]. Older children understand that this is only required when one has a joint commitment with one’s partner: Gräfenhain, Behne, Carpenter, and Tomasello [27] showed that 3-year-old and older children understand that when acting together, but not when simply acting in parallel, both partners are obligated either to continue acting or to take leave of the activity in some way. And Hamann, Warneken, and Tomasello [28] have shown that 3-year-olds understand that if they get access to their part of a joint reward earlier than their partner, they should continue acting until their partner also gets access to his/her reward. Together, these findings suggest that already by 3 years of age, children have an understanding of some of the most basic obligations engendered by joint commitments to act together.

But in adults, at least, these obligations can go much further than waiting for and helping one’s partner. For example, adults can be surprisingly loyal to their joint action partners. Especially after having acted together for some time, adults’ sense of solidarity can become so strong that they might occasionally cover for a lazy partner who does not fulfill her role [29], [30], or cover up for bad (e.g., cheating or unlawful) behavior by a partner, for example, to protect social relationships [31]–[33]. Not only do adults cover for, or cover up this type of behavior, but at the end, they often share the rewards equally even with partners who did not contribute equally to the outcome (e.g., when all the team members – and even the fans – of a winning soccer team are allowed to claim that they won the game).

We know very little about whether young children, like adults, go beyond waiting for and helping a joint action partner who stops acting. There is some research on relevant topics outside the context of joint action. For example, we know that preschoolers grow increasingly skillful at telling lies to protect another person’s feelings [34], [35]. Further, research on young children’s sense of distributive justice has shown that young children are more likely to distribute resources fairly with familiar persons (e.g., friends) than with unfamiliar or unpopular partners [36], [37], and that they share resources fairly when both partners have contributed equally to a joint goal [38], [39]. However, with the exception of these last two studies, these things have not yet been investigated within joint vs. non-joint action contexts.

In the current study we sought to fill this gap by investigating whether young children would show a similar sense of the various obligations that follow from a joint commitment as shown by adults. We therefore engaged children in a game that they played either jointly with a partner or individually, that is, in parallel to another player. The game’s goal was to assemble a puzzle, for which players were promised a reward. In the course of the game, we confronted children with several unexpected events and assessed whether children reacted differently to these events depending on the respective play context. The list of unexpected events was certainly not an exhaustive one, but rather a non-incidental selection of events that potentially perturb joint actions and challenge the partners’ sense of joint commitments to act together [15], [22]–[24]. In particular, in Study 1, we investigated whether, along with waiting for and helping their joint action partner, children would also be willing to take over the partner’s role when she was reluctant to fulfill it, to cover up for the partner’s cheating behavior, and to share the reward at the end fairly with her even though she had not contributed equally to the joint action. If young children’s reactions to these events differed systematically when acting together with a joint partner as compared with when acting alone, this would indicate a much richer and more sophisticated understanding of joint activities than previous research has thus far suggested. Given the findings of previous studies that 3-year-old children readily engage in cooperative activities [27], [28], we tested 3-year-old children.

## Study 1

In Study 1, using a between-subjects design, children either agreed to play a puzzle game with a play partner and then completed the puzzle together with the partner (*collaborative condition*), or else children were encouraged to play the game alone, and completed their own puzzle while another player played the game in parallel, on a separate, identical puzzle nearby (*individual condition*). Children’s play partner in both conditions was a puppet operated by an adult experimenter. A puppet was used to provide children with a play partner who was on a similar social level to themselves. Based on previous studies [40], [41], we expected children to interact with a puppet more informally than with an adult partner and also to feel less inhibited about tattling on a puppet’s transgressions than on those of an adult.

The goal of the game was to complete the puzzle in order to receive a reward. On the way to achieving this goal, five events unfolded. First, when the players had to walk to a different corner of the room to fetch something for the game and the puppet stopped walking, we assessed whether children would adapt their behavior to this interruption of the walk (e.g., by waiting for the puppet). Second, when the puppet accidentally caused damage, we assessed whether children would help her repair the damage and then later try to cover up for her behavior by not indicating her as the source of the damage when questioned by the experimenter. Third, after the puppet cheated by stealing some puzzle pieces to complete the game early, we assessed whether children would cover up for her behavior by not tattling on her when later questioned by the experimenter. Fourth, when the puppet was reluctant to fulfill her role, we assessed whether children would take over her role and complete it for her. Finally, at the end we assessed whether children would distribute a reward equally between themselves and the puppet, despite the difficulties she caused throughout the procedure.

If children understood that agreeing to act together engenders certain obligations whereas participating in individual activities does not, children should react differently in each of the five tests in the two conditions. In particular, we expected more children in the collaborative than in the individual condition 1) to adapt their behavior to an interruption of the activity, 2) to help the partner repair the damage she caused, 3) to cover up for the partner’s deviant behavior, that is, not to indicate the partner as the source of the damage and not to tattle on her cheating behavior (despite the fact that in both conditions children benefitted equally from this cheating behavior), 4) to take over the partner’s role when she was reluctant to fulfill it, and 5) to share the reward fairly with the puppet.

### Materials and Methods

#### Ethics statement

The studies were approved by the Max Planck Institute for Evolutionary Anthropology Child Subjects Committee. They were done with the written informed consent of the children’s parents, and in accordance with all applicable laws and rules governing psychological research in Germany.

#### Participants

Thirty-six 3-year-old children participated in the study (18 girls; mean age = 3;6;02, range = 3;4;11–3;7;29). Children (in both studies) were recruited from a database of parents who had agreed to participate in studies of children’s social-cognitive development.

Families were from heterogeneous socioeconomic backgrounds in a middle-size city in Germany. The majority of children regularly attended day care centers (86%) and had siblings (62%, of which 57% had younger siblings, 29% had older siblings, and 14% had both younger and older siblings). Children received a small gift at the end of the test session.

#### Materials

Children played a puzzle game with a large, child-like puppet (height 50 cm) with legs and a moveable mouth and hands. For the girls the puppet was introduced as a girl and for the boys as a boy (for the sake of convenience, we will refer to the puppet as ‘she’ throughout).

**Figure 1:**
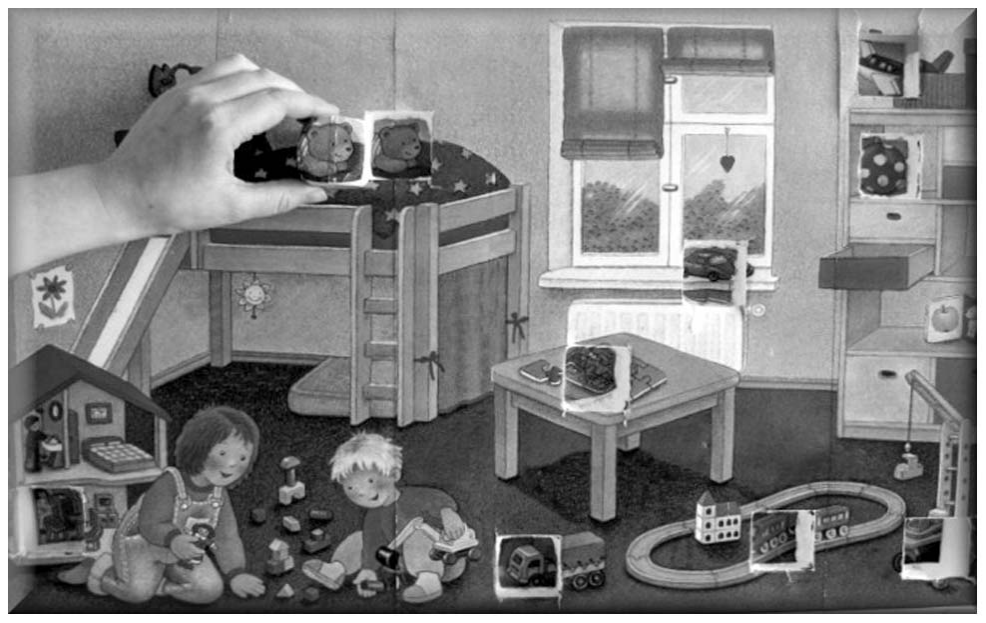
Puzzle board with the scene of a child’s room pasted on top. Nine wooden cubes could be placed into the squares with matching toy pictures on them. In the individual condition, each player played on a separate but identical puzzle board.

The goal of the puzzle game was to complete the puzzle in order to receive a reward. The puzzle was a Styrofoam board (length×width×depth: 72×44×5 cm) with a scene of a child’s room with various toys in it pasted on top. Several square holes (4 cm^3^) were cut out of the Styrofoam over pictures of toys, and the toys’ pictures were pasted to the bottom of the holes. Into these holes could be placed a series of nine wooden cubes with matching toy pictures on them (see Figure 1).

**Figure 2:**
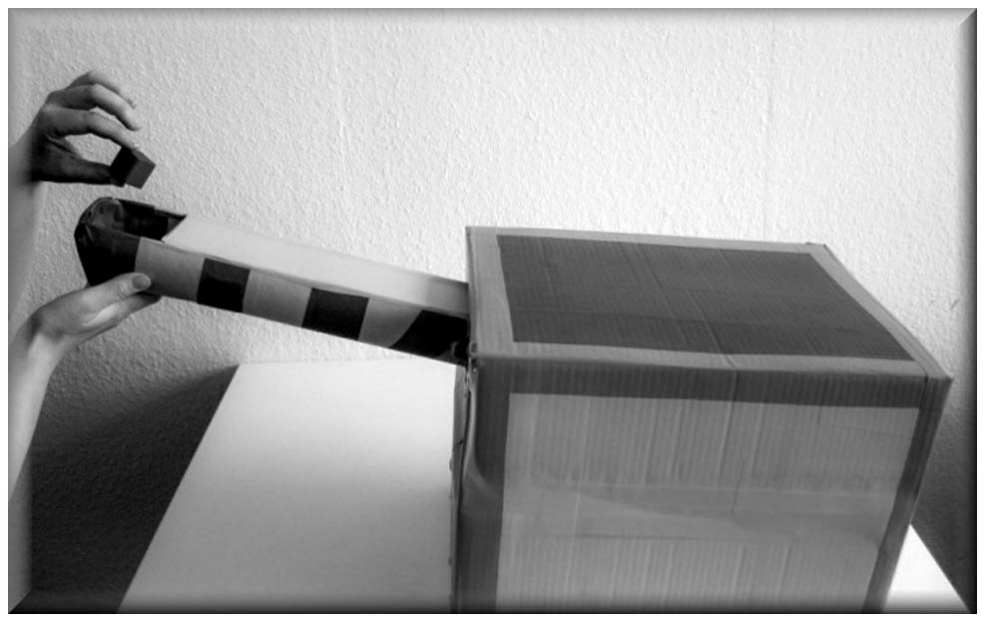
Box containing the puzzle pieces with a moveable chute at one end. Players let two small blocks go down the chute to get access to the puzzle pieces.

At the start of the game, these puzzle pieces were contained in a box (38×30×26 cm) with a moveable chute at the end (see Figure 2). It was explained to children that only the experimenter could retrieve the puzzle pieces (from a door on the back side of this box). To receive each puzzle piece, players had to place two small, colored, wooden blocks (2.5 cm^3^ each) onto the chute and raise the chute so the blocks would fall down into the box.

In the collaborative condition (see below), players played together on the same toys (i.e., there was only one puzzle board and one chute). In each trial, each player received one block of a different color to place onto the chute. In the individual condition, in contrast, there were two identical puzzle boards and chutes and players played with them separately to emphasize the individual nature of the game. In each trial, each player therefore received two blocks of the same color (with a different color for each partner) to place onto the chutes.

The session was recorded by four cameras fixed at the four corners of the testing room.

#### Design and procedure

Children were randomly assigned to one of two conditions in a between-subjects design. Thus, 18 children were assigned to the collaborative and 18 to the individual condition. Gender was approximately evenly distributed between conditions. The sequence of the five tasks was the same for every child. Two adults administered the test sessions, each performing a fixed role. Both were naïve regarding the hypotheses of the study. The experimenter directed the test session and asked the test questions. The assistant operated the puppet.

Children were tested individually in a child studies laboratory in a testing session lasting approximately 45 minutes. After a brief familiarization period with the experimenter, the puppet was introduced to the child. The experimenter interacted with the puppet as if she were another familiar play partner on a similar social level as the child and encouraged the child to play with her. Children were then led to the testing room. Parents were not present in the room but watched the scene through a one-way mirror. Both children and parents had agreed on the parents not being present before the test session started. Children were not aware that their parents were watching them.

The two experimental conditions differed in how the puzzle game was initiated (i.e., with or without an agreement to play together) and how the players played the game (i.e., jointly or in parallel). The procedure of the five subsequent tests was identical for all children in both conditions. The following section describes the game and each test in detail.

#### Demonstration

After a brief familiarization phase in the test room, the experimenter introduced the puzzle game. Sitting behind the puzzle board(s) facing the players, she showed the board(s) to the players, encouraged them to label the depicted objects, and drew the players’ attention to the missing pieces of the puzzle board. She announced that each player would receive a reward when the puzzle(s) were completed. To demonstrate how players could get access to the missing puzzle pieces, she presented the chute(s) and demonstrated how to operate it. She took one of the colored blocks, counted “One, two, three!” and raised the chute to let the block slide down into the box. She repeated this with a second block. She then opened a door at the back of the box, retrieved one puzzle piece and put it into the appropriate location on the puzzle board. She emphasized that she would provide players with two blocks in each round and that they would only receive one puzzle piece at a time (i.e., in the collaborative condition a total of two blocks and one puzzle piece and in the individual condition two blocks and one puzzle piece each). Note that in the individual condition, the experimenter provided each player with separate toys. She therefore first demonstrated the game to the child and then turned away toward the puppet and repeated the demonstration for her, to emphasize the individual play context (thus in this condition children could potentially watch a second demonstration of the game).

#### Initiation of the game

The goal of the initiation phase was to establish the respective play context. Thus, in the *collaborative condition*, the puppet invited the child to play together with her: She asked, “Will you play with me?”, awaited a verbal (e.g., “yes”) or nonverbal (e.g., nod) agreement from the child (thus establishing the joint commitment), and emphasized that they now would play this fun game together. All children readily agreed. The puppet and the child then jointly played the game for three rounds. That is, after the experimenter provided the players with the blocks, the puppet coordinated her actions with the child (e.g., taking turns putting blocks in the chute and encouraging the child to raise it together to let the blocks slide into the box), and conferred with the child about where to put the next puzzle piece. At the end of each round, the puppet looked at the child and said, “We did a good job!” In the *individual condition*, the experimenter announced that each player would now play on his or her own toy and the puppet additionally announced that she would now play this fun game. In the following three rounds, the puppet played individually by not coordinating her actions with and not attending to the child, and by not talking to the child while searching for where to put the new puzzle piece. However, the puppet talked to herself from time to time, resulting in a similar amount of speech as in the collaborative condition. She did all this in an unhurried, relaxed manner, so that there was no suggestion of a competition. In addition, to avoid the possibility of one of the players finishing the puzzle much earlier than the other (and so that the tests could be administered as planned), the experimenter inconspicuously provided the players with the blocks at at least similar points in time. At the end of each round (i.e., after both the child and the puppet had put their block in the puzzle), the puppet said, “I did a good job!” This was done to attract the child’s attention to the puppet’s activities even though she was playing individually. For each of the following five tests, the procedure was identical in both conditions.

#### 1. Interruption test

After three rounds of play, the experimenter announced that there were some blocks missing and that they needed to find them before they could continue playing. She looked around, saying that there should be some more blocks somewhere in the room. At this point, the puppet turned around in the direction of two identical containers (height×diameter: 15×13 cm each) situated in another corner of the room (at a distance of about 3 m diagonal from the play area) and announced that there should be some more blocks over there. She then started walking in the direction of the containers. However, midway to the containers, she suddenly stopped walking to fix her cap, which had fallen off onto the floor, calling attention to her stopping by exclaiming, “Oh no, my cap!”. The puppet then distractedly took 15 seconds to replace her cap (the end of this and all other response phases was signaled by the experimenter, who inconspicuously cleared her throat). Then she started walking again, took one of the containers and returned to the experimenter. Note that throughout this whole test, the puppet (and the experimenter) never encouraged children to accompany her to the containers, and the puppet did not attend to children. Still, all but three children in the individual condition also walked over to the other corner, got one of the containers, and handed it over to the experimenter to continue playing the game. The three children who did not start walking were later excluded from the analysis of this test (see below).

#### 2. Damage test

The experimenter always first took the container the child had brought back and then reached for the puppet’s container. As the puppet moved her arm back after handing her container to the experimenter, she accidentally knocked over the puzzle board (i.e., the communal board in the collaborative condition and her own board in the individual condition), making the puzzle pieces fall out of the board. She exclaimed, “Oops, oh no!” and then kept sitting in front of the board, looking at the board with her hand held in front of her mouth. Until this point, from the moment she had received the container from the puppet, the experimenter had pretended to be distracted by something behind her back. She now turned back to the players, expressed surprise at the fallen board, and neutrally asked the child two questions: 1) “What happened, [child’s name]?”, and 2) “How did it happen, [child’s name]?”. She gave children 10 seconds to respond after each question and always asked both questions irrespective of the child’s responses and of whether or not children were occupied helping the puppet repair the damage (e.g., by picking up the puzzle board and collecting and replacing the puzzle pieces back into the board). To finish the test, the experimenter then announced that this was nothing to worry about and repaired the damage if children had not already done so.

#### 3. Cheating test

To re-establish the respective play context, the players then played three further rounds of the game according to the condition. In addition, children were reminded of the rules of the game to set up the following test. That is, after the experimenter had announced that there were only a few more puzzle pieces missing and that players would soon be able to receive their reward, to speed things along the puppet asked the experimenter whether she could give them more than one puzzle piece this time. The experimenter said no and emphasized that she would only provide one piece. When there were only two puzzle pieces missing (in the individual condition, the assistant made sure that this was also the case for the child’s board), the experimenter inconspicuously placed the last two puzzle pieces next to the chute(s) and pretended to be distracted by something behind her. The puppet spotted the last puzzle pieces, said, “Oh, there are the last two puzzle pieces! If I took them, it would be cheating but still…”, then took them and put them into the board(s) (i.e., in the collaborative condition she put them into the communal board and in the individual condition she first put them in her board and then repeated this for the child’s board, ensuring that in both conditions children benefitted equally from the puppet’s cheating behavior). The puppet finally announced that the puzzle(s) were completed and that now the experimenter could provide the reward. The experimenter turned around, expressed surprise at this, and neutrally asked the child three questions: 1) “What happened, [child’s name]?”, 2) “How did it happen, [child’s name]?”, and 3) “Did anyone cheat?”, looking only at the child. Again she always asked all three questions and gave children 10 seconds to respond after each question. While the experimenter was asking the child these questions, the puppet stayed slightly turned away from the child and pretended to be distracted by her cap, so that children would not feel intimidated by her. Then, to resolve the situation, the puppet admitted that she had taken the last puzzle pieces and apologized for her behavior.

#### 4. Tidying up test

Since the puzzle was now completed and the game was thus almost finished, the experimenter announced that before getting the promised reward, the blocks first had to be tidied up. She therefore took 14 blocks (7 of each color) out of the chute(s) and put them, clumped together, between the players. Next, she took out the two identical containers (previously used in the Interruption and Damage tests) and repeated, “Before receiving the rewards, the blocks have to be tidied up. They have to go in here.” She put one container in front of each player and then again she turned away, pretending to be distracted by something behind her. The puppet, however, lazily did not complete her role: She put one of her blocks into her container, then picked up another, but then let it fall to the floor, saying, “Oh no, that’s no fun!” She then pretended to be distracted by her cap for 20 seconds. If the child had not finished tidying up all the blocks by the end of those 20 seconds, the experimenter turned around, encouraged the puppet to tidy up once more and again turned her back to the players. The puppet then repeated her unwillingness to do this and pretended to be distracted for another 20 seconds. If the child had not tidied up all the blocks by then, the experimenter firmly asked the puppet to finish the task so she could present the rewards and she did so.

#### 5. Sharing test

To finish the session, the experimenter announced that before receiving the final big reward, she had a small reward for the previous tidying up. She put seven stickers in front of the child and provided two small, differently colored containers (height×diameter: 10×8.5 cm), placing one in front of the child and one in front of the puppet and explaining that one was for the child and one was for the puppet. She then told the child, “The stickers for the puppet have to go in this container and the stickers for you have to go in that container. Would you please divide them up?” She reminded the child that the stickers were a reward for the tidying up and turned her back to the players. She repeated this instruction during the response phase if necessary (e.g., when encouraging the child). While the child distributed the stickers, the puppet was turned away from the child pretending to be distracted by her cap. After the child had finished, the players were given the stickers and the child received the final reward in the form of a small toy to take home.

#### Coding and reliability

We were mainly interested in whether children responded to the various tests differently depending on the respective (collaborative vs. individual) play context. The supporting information provides a detailed description of the coding criteria: Table S1.

#### 1. Interruption test

In this task, the puppet started walking to two containers in another corner of the room. Midway to the containers, she unexpectedly stopped walking for 15 sec and we coded whether children adapted their behavior to this interruption (by either waiting for her to continue walking or by helping her), or whether they continued walking alone and brought one of the containers back to the experimenter.

#### 2. Damage test

When handing her container to the experimenter, the puppet accidentally knocked over the puzzle board. We were mainly interested in 1) whether children spontaneously helped repair the damage (e.g., by picking up the puzzle board or collecting and replacing the puzzle pieces), and 2) when children started helping, that is, whether they started to repair the damage before the experimenter turned around and asked any questions, after question 1, or after question 2.

In addition, when the experimenter asked what had happened and how it had happened, we coded whether or not children indicated the puppet as the source of the damage or whether they avoided indicating her. Thus, for each question, we coded whether children showed one of the following types of behavior: a) indicating the puppet either verbally or nonverbally; b) responding uninformatively either verbally or nonverbally (e.g., responding that the board had fallen over without indicating who had caused the damage); or c) not clearly responding to the question.

#### 3. Cheating test

The puppet cheated in order to speed things along. When questioned about the event by the experimenter, we were interested in whether or not children would tattle on the puppet’s cheating behavior. The coding was identical to that for the questions in the Damage test. In addition, children were directly asked whether someone had cheated. We coded whether children admitted that there had been cheating (verbally or nonverbally), denied the cheating (verbally or nonverbally), or whether they did not respond to this question at all.

#### 4. Tidying up test

In this test, players were presented with two containers and were encouraged to tidy up 14 blocks, but the puppet did not complete her role. We were mainly interested in whether children picked up the slack for the lazy puppet and took over her role as well as their own. Thus, we coded whether children tidied up a) all blocks, b) at least half of the blocks, or c) less than half of the blocks (the exact number of blocks could not be coded reliably from recordings since children frequently picked up and put in several blocks at a time). We also realized during coding that which container(s) children put the blocks into might be interesting since the experimenter had not instructed the players about which container(s) they should put the blocks into and did not watch the tidying up. Children could thus easily indicate their own contribution to the tidying up by putting all the blocks they tidied up into their own container. Alternatively, children could put blocks into both containers, hindering the experimenter from knowing which blocks each player had tidied up and/or giving the puppet (undeserved) credit for cleaning up all her blocks. We therefore coded into which container children sorted the blocks: only their own container, only the puppet’s, or both containers.

#### 5. Sharing test

Children were presented with seven stickers, which they were told to distribute into two containers based on the players’ previous tidying up performance. We coded the number of stickers children put into their own and into the puppet’s container.

#### Behavior across tests

Children participated in a variety of tests. We were therefore interested in whether children’s behavior was consistent across the tests, for example, whether children who waited for the puppet in the Interruption test later also took over her part in the Tidying Up test. We therefore binarily coded the main measures for each child in each test. In particular, we coded whether or not children waited or helped the puppet (Interruption test), were reluctant to indicate or to tattle on the puppet (Damage and Cheating tests), cleaned up all blocks (Tidying Up test), and shared the stickers more or less equally (i.e., gave the puppet at least 3 out of 7 stickers; Sharing test).

Children’s behavior was coded from the video recordings by the first author. A second coder who was blind to the hypotheses of the study coded a random selection of the sample (14 children, 38% of the sample) resulting in good inter-observer reliability: Interruption test: 86% agreement between coders, κ = .71 for the waiting measure; Damage test: 100% agreement, κ = 1 for the helping measure; Damage and Cheating tests, 85% agreement, κ = .75 for children’s responses to the experimenter’s questions; Tidying Up test: 93% agreement, κ’s = .85 for both tidying up the blocks and children’s use of containers; Sharing test: 93% agreement, κ = .89 for children’s distribution of the stickers to the players).

### Results and Discussion

#### Preliminary analyses

Preliminary analyses revealed no effect of gender on any of the measures. This factor was therefore collapsed for the following analyses. In the Damage and Cheating tests, children were asked two different questions by the experimenter (what had happened and how it had happened). Overall, children responded actively in the majority of response phases by either indicating the puppet or by responding uninformatively to the experimenter’s questions (*M* = 67% of response phases). The effects reported below are thus not due to a general reluctance to respond to the experimenter’s questions. We further found no systematic differences in children’s responses to the two questions in either test (Wilcoxon signed ranks test, *p*’s >.51). For each test, we therefore collapsed children’s responses across the two questions (i.e., what had happened and how it had happened), coding whether children indicated the puppet at least once.

Each test consisted of only one trial per child, so we conducted Chi-square tests. All *p* values reported are two-tailed.

#### 1. Interruption test

We compared the number of children who adapted their behavior to the puppet’s interruption in walking (by either waiting for the puppet or by helping her) to the number of children who continued walking without reacting to the puppet’s behavior as a function of condition. Three children did not start walking to fetch the containers but remained sitting with the experimenter (all in the individual condition). These children were not included in the following analysis. The results revealed that significantly more children in the collaborative condition (67%) reacted to the interruption than children in the individual condition (20%; Chi-square test, χ^2^ (1, *N* = 33) = 7.19, *p*<.05; Odds ratio = 8). Thus, when children had agreed to play together with the puppet, they were more likely to adapt their behavior to the (unexpected) behavior of the puppet than when they had played individually. This suggests that 3-year-olds understand that when they are jointly committed to play together, partners should coordinate their behavior with each other not only during the actual joint activity (e.g., by coordinating where to put the puzzle pieces) but also when they are confronted with unexpected events related more tangentially to the main joint activity. This finding is thus reminiscent of the commitments that adults feel in Gilbert’s famous example of two people ‘taking a walk together’ [23].

#### 2. Damage test

The puppet caused (accidental) damage and we were interested in whether children would spontaneously help repair the damage. Although twice as many children in the collaborative condition (56%) as in the individual condition (28%) helped at some point during the response phases, this difference was not significant (Chi-square test, *p*>.17; Odds ratio = 3.25). However, when we considered when children started to help the puppet, we found that 44% of children in the collaborative condition helped even before the experimenter had turned around and noticed that damage had occurred, whereas no child in the individual condition helped at that early point in time. This difference reached significance (Chi-square test, χ^2^ (1, *N* = 36) = 10.29, *p*<.01; Odds ratio = 15.5), (note that for calculating the odds ratio with a cell of zero, a constant of .5 was added to each cell).

Next, we were interested in whether children indicated the puppet as the source of the damage when questioned by the experimenter, in particular, whether children in the collaborative condition were especially reluctant to indicate the puppet. We thus compared the number of children who indicated the puppet at least once with the number of children who never indicated her (by responding uninformatively or not at all). We found that overall, only a minority of children indicated her, and they did so equally in both conditions (28% of children in the collaborative and 33% of children in the individual condition, Chi-square test, *p* = 1; Odds ratio = 0.77).

Since we assessed two different types of behavior after the puppet had caused damage, we were interested in whether children’s behavior was consistent when reacting to the damage, for example, whether children who first supported the puppet by helping repair the damage would also be reluctant to indicate her as the source of the damage when later questioned by the experimenter. In the next set of analyses, we therefore tested whether children’s helping behavior was related to their indicating the puppet. We found that in the collaborative condition, children’s helping behavior was linked (negatively) to their indicating (Chi-square test, χ^2^ (1, *N* = 18) = 8.65, *p*<.01; Odds ratio = 2.2). In particular, all 10 of the children who helped the puppet did not indicate her when later questioned by the experimenter, and none of the five children who indicated the puppet had previously helped her repair the damage. No such relation was found for children in the individual condition (Chi-square test, *p*>.6; Odds ratio = 0.4): Only four children in this condition both helped and declined to indicate the puppet.

Thus, children did not differ between conditions in their reluctance to indicate the puppet when she had caused damage. However, they did differ in their helping behavior in that those children who had previously played together with the puppet spontaneously helped repair the damage before the experimenter had even noticed that the damage had occurred. This finding must be taken with some caution, however, as it is possible that children in the collaborative condition helped more quickly because the damage had occurred to the puzzle that they themselves were working on. Still, it is interesting that it was these particular children who later were reluctant to indicate the puppet as the source of the damage. Children who had played individually, in contrast, did not show such consistent, systematic behavior. Thus, it appears that 3-year-old children understand that when they are jointly committed to act together, partners should help each other when necessary, thereby ensuring that the joint activity could be continued.

#### 3. Cheating test

Children had witnessed the puppet cheating in order to receive the game’s final reward earlier than expected. Upon being questioned by the experimenter, we were interested in whether children differed in tattling on the puppet’s behavior as a function of condition. We found that, as in the Damage test, only a minority of children tattled, and they did so equally in both conditions (28% of children in each condition, Chi-square test, *p* = 1; Odds ratio = 1). When children were directly asked whether someone had cheated in question 3, children also reacted equally in both conditions: 56% of children in each condition admitted that there had been cheating (Chi-square test, *p* = 1; Odds ratio = 0.97). Thus, children were generally reluctant to tattle on the puppet’s transgression, irrespective of whether or not they had been playing together with her (see below for further discussion of this finding).

#### 4. Tidying up test

Players were asked to tidy up some blocks before receiving the game’s final reward; however, the puppet was lazy and did not complete her role. We were interested in whether or not children picked up the slack for her and performed her role differently as a function of condition. We found that 78% of children in the collaborative condition tidied up all blocks whereas only 50% of children in the individual condition did so. This difference was not significant (Chi-square test, *p*>.16; Odds ratio = 3.5). However, children in the collaborative condition cleaned up all blocks significantly more often than expected by chance (binomial tests of the binarily-coded data against the fixed value of.5, *p*<.05), whereas children in the individual condition were at chance (*p* = 1).

Next we looked at which containers children used when tidying up: only one container, (either their own or the puppet’s), or both containers. One child in the control condition had to be excluded from analyses because she immediately cleaned up all her blocks and pushed them over to the experimenter without using any container. We found that twice as many children in the collaborative condition (72%) as in the individual condition (35%) divided the blocks into both containers. Given that the majority of children who only used one container used their own (80% in the collaborative and 100% in the individual condition), we collapsed the data of children using one container and binarily coded whether children used one or both containers. The Chi-square test between conditions revealed a significant difference (χ^2^ (1, *N* = 35) = 4.8, *p*<.05; Odds ratio = 4.77). Finally, we compared whether children used both containers more often than expected by chance. We found that children did not use both containers more often than expected by chance in either condition (binomial tests, *p*>.09). Again, since we assessed two different types of tidying up behavior (the number of blocks and the containers children used), we were interested in whether children’s behavior was consistent. We found that the number of blocks children tidied up was significantly positively correlated with the containers they used to tidy up, both across conditions and within the collaborative condition separately (across conditions: *rho* = .55, *p*<.01, *N* = 35; collaborative condition: *rho* = .58, *p*<.05, *N* = 18; for the individual condition it was marginally significant: *rho* = .45, *p = *.07, *N* = 17). Thus, again we see consistency in children’s behavior, especially in the collaborative condition.

In sum, although there was no significant difference in how many children tidied up all the blocks, children in the collaborative condition tidied up significantly more blocks than expected by chance, and they were more likely to use both containers to tidy up the blocks. It is unclear at this point why these children used both containers and there are several possible explanations. One could argue that children merely used both containers because they needed more space to store the blocks. This is unlikely, however, given that half of the children in the individual condition tidied up all blocks but mainly used their own, that is, only one container. Alternatively, children may generously have tried to give the puppet (i.e., their play partner) credit for her previous play behavior by taking over her role in the activity. Or children may have tried to hide the puppet’s lazy behavior by deceiving the experimenter about who did what when presenting two containers filled with blocks. Future research may look at what motivation drove children in the collaborative condition (but not in the individual condition) to show this particular type of behavior. Together, the findings of the Tidying Up test suggest that children who had played jointly with the puppet were slightly more likely to take over her role when necessary than children who had played individually. Three-year-old children thus may be beginning to understand that when jointly committed, partners need to support each other, even to the extent of taking up the slack for partners who do not complete their role. Children who had played individually, in contrast, were not as ready to take over more of the task than they were expected to.

#### 5. Sharing test

Children were encouraged to distribute seven stickers into two containers (one for each player) as a reward for the players’ tidying up behavior in the Tidying Up test. There was no difference between conditions in the number of stickers children distributed to themselves vs. the puppet (mean number of stickers children distributed to themselves in the collaborative condition: *M* = 4.1, *SE* = .36; in the individual condition: *M* = 4.9, *SE* = .42; independent samples *t*-test, *t*(34) = −1.5, *p* = .14). However, when we compared the mean number of stickers children distributed to themselves with the chance level of 3.5 (i.e., half of the stickers), we found that children in the individual condition distributed significantly more stickers to themselves than expected by chance (one-sample *t*-test, *t*(17) = 3.4, *p<*.01), whereas children in the collaborative condition did not differ from chance level (*p*>.11). Thus, in the collaborative condition, children distributed the sticker rewards equally, even though their partner had not ‘pulled her weight’ during the previous Tidying Up test. This finding extends previous research on young children’s distributive justice in collaborative activities [38], [39], [42] by showing that children distribute the reward fairly even when the partners did not contribute equally to the joint outcome.

#### Behavior across tests

To test whether children’s behavior was consistent across the five different tests, we conducted Cochran’s Q tests, for each condition separately. This analysis tests the hypothesis that children’s behavior does not differ systematically across the tests, in other words, it tests whether children who had reacted to the puppet’s interruption in the Interruption test, for example, later helped the puppet in the Damage test and took over her role in the Tidying Up test. Results revealed that in the individual condition, children behaved rather inconsistently across tests (Cochran’s Q = 12.41, *N* = 18, *df* = 4, *p*<.05; note that for this analysis, the three children who did not start walking to fetch the container in the Interruption test were included). In contrast, children’s behavior in the collaborative condition was more consistent and systematic across the five different tests (Cochran’s Q = .56, *N* = 18, *df = *4, *p* = .99). Thus, although children’s behavior did not differ significantly between conditions in all of the tasks, children’s performance in the collaborative condition was systematically partner-directed and held even across events with negative behavior on the part of the partner (e.g., the puppet cheating or being lazy). This indicates that the initial joint commitment was quite robust against disturbances whereas no such stabilizing element seemed to be present in the individual condition.

Taken together, the findings of Study 1 suggest not only that 3-year-old children understand that agreeing to act together obligates partners to continue acting until the shared goal is achieved [27], [28] but also that children of this young age are starting to understand a range of consequences that joint commitments may entail: They wait for and help their partner if necessary, and support her and tend to share the activity’s outcome equally with her (even if she put in less work). This suggests an even more sophisticated understanding of what it means to act together than suggested by previous research.

One surprising finding, however, was that most children in the study were reluctant to tattle on the puppet, even when they had previously played the game individually. We had expected children in the individual condition to feel less obligated to protect the puppet than children who had a joint commitment to play together with her. Similarly, in the Sharing test, although we found that children who had played together with the puppet shared the reward equally with her, whereas children who had played individually distributed more stickers to themselves, this difference was relatively weak in that the conditions did not differ significantly from each other. One possible explanation for both of these findings is that the puppet was present when children were questioned by the experimenter in the Cheating test and while they distributed the stickers in the Sharing test, so children might have felt constrained or inhibited by her presence and ability to observe their responses. We therefore conducted a follow-up study, in which we administered some of the tasks from Study 1 (the Cheating, Tidying Up and Sharing tests) with the puppet absent when children were asked about the cheating situation and while they distributed the stickers. The goal of Study 2 was thus to investigate whether children would be more likely to tattle on the puppet or to distribute the rewards differently in a more anonymous setting.

## Study 2

In Study 2, we first had 3-year-olds engage in collaborative or individual play as in Study 1. Then we administered only three of the five tests – the Cheating test, the Tidying Up test and the Sharing test. In contrast to Study 1, during the response phase of these tests the puppet was distracted in a different corner of the room when children were asked the test questions in the Cheating test and when they had to distribute the stickers in the Sharing test. We thus expected children to feel less observed and less overheard by her during these test phases. In addition, to reduce noise in the Tidying Up test (because for some of the children the puppet ended up tidying her blocks), in this study the experimenter finished tidying up the last blocks herself if necessary, instead of making the puppet do it. We also increased the number of stickers children had to distribute in the Sharing test (9 instead of 7 stickers) and introduced different qualities of stickers (i.e., some ‘fancy’ and some ‘boring’ stickers) to increase children’s motivation to consider which sticker they distributed to which player [43]. Again we predicted that children’s behavior would differ as a function of play context (i.e., collaborative vs. individual play). In particular, we expected that children who had agreed to play together with the puppet would be more reluctant to tattle on the puppet after her cheating and more likely to distribute the sticker rewards fairly between the players than children who had played the game individually.

### Materials and Methods

#### Participants

Thirty-six different 3-year-old children participated in the study (18 girls; mean age = 3;6;08, range = 3;2;25–3;11;10). The majority of children regularly attended day care centers (97%) and had siblings (77%, of which 78% had younger and 22% had older siblings). Six additional children were tested but could not be included in the final analyses because of uncooperative behavior (4 children) or experimenter error (2 children).

#### Materials

The same materials were used as in Study 1, except that in the Sharing test, we presented children with stickers of different quality: four ‘fancy’ stickers and five ‘boring’ stickers differing in size, texture and the type of picture depicted on them (e.g., big, shiny, textured pictures of animals, cars or fairies vs. small, flat pictures of lady bugs and clovers).

#### Design and procedure

The sequence of the three tasks was the same for every child. Two different teams of experimenters carried out the test sessions in two different cities (both trained and supervised closely by the first author).

The procedure of the warm-up, demonstration, and initiation phase was the same as in Study 1. Half of the children agreed to play the game together with the puppet and played with her on a single puzzle board and chute *(collaborative condition)*, whereas for the other half of children, parallel play was initiated and players played on their own puzzle boards and chutes *(individual condition)*.

#### 1. Cheating test

As in Study 1, children were reminded that players would only receive one puzzle piece at a time, but the puppet cheated by taking the last two pieces and putting them into the puzzle board(s). However, differently from Study 1, after the puppet announced that the puzzle was completed, she went to a different corner of the room (about 2.50 m away from the play scene) to drink some juice (with her back turned to the child). The experimenter turned around, expressed surprise at the completed puzzle and neutrally asked the child the same three questions: 1) “What happened, [child’s name]?”, 2) “How did it happen, [child’s name]?”, and 3) “Did anyone cheat?” During the whole response phase, the puppet stayed in her corner with her back turned pretending to drink. The puppet returned to the play scene after the experimenter called her back, and apologized for her behavior.

#### 2. Tidying up test

The procedure of this test was identical to that of Study 1, with the exception that if the child had not tidied up all the blocks by the end of the test phase, the experimenter finished tidying up herself, expressing mild annoyance by saying, “Well, then it will be me tidying up the blocks.”

#### 3. Sharing test

Differently from Study 1, the puppet was not present when the experimenter asked the child to distribute the stickers: again she went to another corner of the room to drink some juice (with her back turned). The experimenter put nine stickers in front of the child, remarking on the stickers’ different quality (i.e., preferring the ‘fancy’ to the ‘boring’ stickers) and provided the child with the two containers. While the child distributed the stickers, the puppet stayed in her corner, her back turned to the child.

#### Coding and reliability

The coding procedure was identical to that of Study 1 except for in the Sharing test. In this test, children were presented with nine stickers of different quality and we coded the number and the type of sticker (i.e., the number of ‘fancy’ and ‘boring’ stickers) children put into their own and the puppet’s container. In order to test whether children’s behavior was consistent across tests, we binarily coded whether or not children were reluctant to tattle on the puppet in the Cheating test, tidied up all the blocks in the Tidying Up test, and distributed the stickers more or less fairly (i.e., gave the puppet at least 4 out of 9 stickers) in the Sharing test.

Children’s behavior was coded from the video recordings by the first author. A second coder who was blind to the hypotheses of the study coded a random selection of the sample (14 children, 38% of the sample), resulting in excellent reliability: Cheating test: agreement between coders: 89% of cases, κ = .89 for children’s responses to the experimenter’s questions, Tidying Up test: 100% agreement, κ’s = 1 for both tidying up the blocks and children’s use of containers; Sharing test: 100% agreement, κ = 1 for children’s distribution of the stickers.

### Results and Discussion

Preliminary analyses revealed no effect of children’s gender or the team of experimenters in the two cities on any of the measures. These factors were therefore collapsed for the following analyses.

#### 1. Cheating test

Children had witnessed the puppet cheating to receive the reward earlier than expected and were then asked two different questions by the experimenter (what had happened and how it had happened). Overall, children responded actively in the majority of response phases by either indicating the puppet or by responding uninformatively (*M* = 65% of response phases). The effects reported below are thus not due to a general reluctance to respond to the test questions. There were no systematic differences between the two questions (Wilcoxon signed ranks test, *p*>.78), so we collapsed children’s responses across questions, coding whether children indicated the puppet at least once.

We mainly replicated the findings of Study 1 in that only a minority of children tattled (28% of children in the collaborative condition, 39% children in the individual condition), with no significant difference between conditions (Chi-square test, *p*>.7; Odds ratio = 0.6). When children were directly asked whether someone had cheated in question 3, children again reacted similarly in both conditions: 71% of children in the collaborative condition (out of 17 children; one child could not be coded for technical reasons) and 56% children in the individual condition admitted that there had been cheating (Chi-square test, *p*>.48; Odds ratio = 1.92).

In sum, again children seemed to be reluctant to tattle on the puppet’s transgression, irrespective of whether they had been playing together with her or individually – even though the puppet was across the room with her back turned when children were questioned by the experimenter.

#### 2. Tidying up test

This test was repeated simply in order to set up the Sharing test, but we present the results for comparison with those of Study 1. Regarding the number of blocks that children tidied up, we found that only one child tidied up less than half of the blocks and therefore we collapsed the two coding categories ‘less than half’ and ‘at least half’ of the blocks, to have two categories: ‘all’ and ‘less than all.’ Almost 27% more children in the collaborative condition (94%) tidied up all blocks than children in the individual condition (67%). This difference was marginally significant (Chi-square test, χ^2^ (1, *N* = 36) = 4.43, *p* = .09; Odds ratio = 8.5). Moreover, children in the collaborative condition cleaned up all blocks significantly more often than expected by the chance level of.5 (binomial test, *p*<.001), whereas children in the individual condition did not (*p*>.23).

Regarding the containers children used for tidying up, we failed to replicate the findings of Study 1 in that children were equally likely to use both containers instead of only one of them (50% of children in the collaborative condition and 44% of children in the individual condition used both, Chi-square test, *p* = 1; Odds ratio = 1.25). Children did not use both containers more often than expected by chance in either condition (binomial tests, *p*>.81).

However, again the number of blocks children tidied up was significantly correlated with the containers they used to tidy up, both across conditions (*rho* = .47, *p*<.01, *N* = 36) and in the individual condition (*rho* = .63, *p*<.01, *N* = 18). In the collaborative condition, no significant correlation was found (*rho* = .24, *p = *.32, *N* = 18). This last result, however, was probably caused by the low variability of behavior given that all but one child in this condition tidied up all the blocks.

In summary, whereas the specific results of this study differed somewhat from those of Study 1 in this test, the general pattern of results across studies suggests that there was some tendency for children who had played together with the puppet to be more likely to take over her role and to compensate for her laziness (either by cleaning up more of her blocks or by putting blocks into her container for her) than children who had played individually.

#### 3. Sharing test

Children were encouraged to distribute nine stickers of different quality (4 ‘fancy’ and 5 ‘boring’ stickers) into two containers (one for each player) as a reward for the previous tidying up behavior, while the puppet was away. We found no difference between conditions in the quantity of stickers children distributed to themselves vs. the puppet (mean number of stickers distributed to themselves in the collaborative condition: *M* = 5.4, *SE* = .41; in the individual condition: *M* = 5.7, *SE* = .62, independent samples *t*-test, *t*(34) = −.45, *p* = .66). The same result was found for the quality of stickers children distributed to themselves (mean number out of four ‘fancy’ stickers distributed to themselves in the collaborative condition: *M* = 2.5, *SE* = .28; and in the individual condition: *M* = 2.9, *SE* = .26, independent samples *t*-test, *t*(34) = 1.15, *p* = .26).

In the next step of analyses, we compared the mean (total) number of stickers children distributed to themselves with the chance level of 4.5 (i.e., half of the stickers). We found that in the collaborative condition, children distributed significantly more stickers to themselves than expected by chance (one-sample *t*-test: *t*(17) = 2.2, *p<*.05) and that children in the individual condition tended to do this too (*t*(17) = 1.9, *p = *.06). Children’s fair sharing behavior in Study 1 could thus indeed have resulted from the puppet being present while children shared the stickers. However, when comparing the mean number of ‘fancy’ stickers children distributed to themselves, children in the collaborative condition did not differ from the chance level of two stickers (*p* = .10), whereas children in the individual condition distributed significantly more of the fancy stickers to themselves than expected by chance (*t*(17) = −3.6, *p<*.01).

Thus, although again some of the specific results differed between Studies 1 and 2, the general pattern of results suggests that children who had played together with the puppet distributed the stickers (or just the best, ‘fancy’ stickers) fairly whereas children who had played individually distributed more to themselves than expected by chance. Whether children distributed the stickers based on quantity (Study 1) or mainly on quality (Study 2), this finding corroborates recent findings that 3-year-old children are beginning to consider the value of the resources they distribute [43], and that, especially in joint action contexts, young children are capable of sharing resources equally [39], [42].

#### Behavior across tests

As in Study 1, we tested whether children’s behavior was consistent across the three different tasks. We conducted Cochran’s *Q* tests for each condition separately. Results revealed that children’s behavior was rather consistently partner-directed across the three tasks in the collaborative condition, although in this study it was also consistent (i.e., consistently less-partner-directed) in the individual condition (both *p*’s >.14).

## Analyses across Studies

Some of the results from Studies 1 and 2 were weak or mixed. One limitation of these studies is the relatively small sample size. It often happened that findings went in the predicted direction (with differences of up to 28% of children between conditions) but failed to reach statistical significance, perhaps due to a lack of power. Thus in a final set of analyses, across studies we collapsed the data from the three tests that both studies had in common: the Cheating, Tidying up and Sharing tests. This allowed us to double the sample size (to *N* = 71 or 72, see above). We used binarily-coded data for analyses because some procedural details differed between the studies (e.g., the number of stickers used in the Sharing tests). That is, we binarily coded whether or not children were reluctant to tattle on the puppet in the Cheating test, tidied up all the blocks in the Tidying Up test, and distributed the stickers more or less fairly (i.e., gave the puppet at least 3 out of 7 stickers in the Sharing test of Study 1 and at least 4 out of 9 stickers in Study 2). We therefore could only analyze the main measures of each test.

### Cheating Tests

We found that across studies, the majority of children did not tattle on the puppet and tell the experimenter that she had cheated to gain the reward earlier than expected (i.e., 72% of children in the collaborative conditions and 67% of children in the individual conditions did not tattle). This difference did not reach significance (Chi-square test, *p>*.79; Odds ratio = 1.3). When directly asked by the experimenter in question 3, the majority of children in both conditions admitted that there had been cheating (67% of children in the collaborative conditions and 56% of children in the individual conditions, Chi-square test, *p>*.45; Odds ratio = 1.6). Thus, these findings support the idea that 3-year-old children are already sensitive to norms against tattling at least in the context of a game – they do tattle to some extent about moral transgressions such as destroying someone else’s possessions [41], [44], [45].

### Tidying Up Tests

When analyzing whether children across studies tidied up all or only some/none of the blocks, we found that 86% of children in the collaborative conditions cleaned up all blocks compared to only 58% of children in the individual conditions. This difference was significant (Chi-square test, χ^2^ (1, *N* = 72) = 6.92, *p<*.05; Odds ratio = 4.4).

Again, we compared whether children cleaned up all blocks significantly more often than expected by chance. We found that only children in the collaborative conditions did this (binomial test against the fixed value of.5, *p*<.01); children in the individual conditions did not (*p* = .41). When analyzing which containers children used for tidying up (i.e., both containers or only one of them, mainly their own), we found that 61% of children in the collaborative conditions used both containers compared to 40% of children in the individual conditions. This difference was marginally significant (Chi-square test, χ^2^ (1, *N* = 71) = .3.16, *p = *.09; Odds ratio = 2.4).

We found that the number of blocks children tidied up was significantly positively correlated with the containers they used (i.e., the more blocks children tidied up the more likely they were to use both containers), both across conditions and within the different conditions separately (across conditions: *rho* = .49, *p*<.01, *N* = 71; collaborative condition: *rho* = .34, *p*<.05, *N* = 36; individual condition: *rho* = .55, *p<*.01, *N* = 35). Thus, again we see consistency in children’s behavior.

### Sharing Tests

When analyzing children’s distribution of stickers, we found that 69% of children in the collaborative conditions and 56% of children in the individual conditions behaved unselfishly: they gave at least 3 out of 7 or 4 out of 9 stickers to the puppet. This difference did not reach significance (Chi-square test, *p*>.32; Odds ratio = 1.82). We then compared whether children gave the puppet at least this number of stickers more often than expected by chance. We found that only children in the collaborative condition did so (binomial test against the fixed value of.5, *p*<.05), whereas children in the individual condition did not (*p* = .62).

In sum, we found that children shared the reward for tidying up somewhat unsystematically across studies. Children’s behavior did not differ significantly between conditions; however in two out of the three comparisons to chance in the two studies, as well as in this overall analysis, children in the collaborative condition shared almost equally whereas children in the individual condition did not.

### Behavior across Tests

When comparing the three repeated tests with each other collapsed across studies, results revealed that children’s behavior was rather consistent across the test session in the collaborative conditions, and it was also consistent (i.e., consistently less-partner-directed) in the individual conditions (both *p*’s >.24).

## General Discussion

Joint commitments are the glue that holds joint action together, even when something goes wrong. They create a ‘we-attitude’ – ‘we’re in this together’ – that has a wide range of consequences for participants’ actions which, in adults at least, may go as far as taking over the role of a lazy partner or covering up for a partner’s bad behavior. In the current studies, we found that 3-year-old children understand some of these same consequences as well.

First, we found that after having agreed to play together with a partner and playing with her briefly, children adapted their own behavior to an interruption by the partner (when waiting for the hindered partner in Study 1) whereas children who had played individually did not. This replicates previous findings on young children reengaging or waiting for a collaborative partner when the other suddenly stops acting [3]–[5], [14], [27]. It also extends these findings by showing that children adapt their behavior to that of their partner even when they are confronted with unexpected events that are related more tangentially to the main joint activity (in this case, fetching further objects to use in the main activity). Thus, young children, like adults, are beginning to understand that collaborative partners are supposed to adapt their behavior to each other even when unexpected events occur in the course of a joint activity [23], [24].

Second, we found that 3-year-old children tended to support their collaborative partner when needed, in various ways. They helped the puppet repair damage she had caused accidentally (as children in the individual condition did as well), but did so more quickly and spontaneously than children in the individual condition (although alternative explanations for this finding are possible). Children even tended to take over the puppet’s role for her when she lazily refused to fulfill it: They tidied up her blocks as well as their own more often than children who played individually, with children in the collaborative condition tending to put her blocks into her container for her instead of putting them all into their own container. Children thus seemed to be willing to put more effort into the joint activity than initially designated to their own role in order to ensure that the joint activity proceeded successfully.

Third, we found somewhat mixed evidence regarding whether children were willing to give more credit (in the sense of rewards for effort) to their partner than perhaps she deserved in the collaborative condition. These findings mirror the mixed findings in research on young children’s prosocial and sharing behavior more generally [36], [37], [39], [46]–[48]. Thus, future research will have to investigate further whether 3-year-old children genuinely understand that, when collaborating, each partner should receive an equal share of the reward, even if they have not put in an equal amount of effort. Another finding was that children were quite reluctant to tattle on the puppet, both when she had caused damage accidentally and when she had intentionally cheated to speed the game along. Even when the puppet was not there to hear, children refrained from tattling on her in both of these situations. Their reluctance to tattle was expected in the collaborative condition, as we know that adults often cover for joint action partners [31]–[33]. However, it is somewhat surprising in the individual condition.

There are several possible explanations for children’s reluctance to tattle. A recent study by Ingram and Bering [44] has shown that in free play situations, 3- and 4-year-olds tattle mainly on physical aggression or property damage and less on conventional transgressions of other children [41], [49]. Children in our studies, however, were asked to tattle on a rule (i.e., conventional) transgression, and importantly one from which they even benefitted (allowing them to receive the game’s final reward earlier than expected). They thus may not have been sufficiently motivated to admit that cheating had occurred. Children might also have refrained from tattling in order to protect the puppet, to allow her to save face. In both conditions, they had played alongside her for some time at this point and thus she was familiar and probably likeable to children. Or children at this age might already be sensitive to more general norms against tattling, and refrained from implicating the puppet for this reason [34], [35]. It is unlikely that they were simply afraid of getting in trouble when admitting the cheating, since the majority of children admitted that there had been cheating when the experimenter directly asked them about this. Future research is needed to investigate under what circumstances preschool children are likely to tattle on another person’s misbehavior.

One might argue that perhaps children in the individual condition understood the context of the game as competitive and that this affected their behavior towards the puppet in the tests. This is unlikely, however, because in her instructions the experimenter emphasized that each player would receive a reward at the end of the game, and the atmosphere and the puppet’s behavior was friendly and non-competitive throughout. Further, the finding that children in the individual condition were reluctant to tattle on the puppet and even supported her to some extent suggests that the puppet was at least familiar and likeable for most children across conditions.

Most children participating in the current studies attended daycares and/or had siblings. Given the low variability of these factors in the samples, we could not analyze whether experiencing regular social interaction with peers in everyday life affected children’s behavior in the tests, for example, by them showing enhanced socio-cognitive skills or more cooperative (or else competitive) motivation [36], [49]–[52]. Future research may examine more systematically whether and how living with siblings or attending daycare affects children’s motivation and ability to engage in cooperative activities.

One open question that remains is how joint commitments come about, and are sustained even in the face of perturbations to the joint action, in young children (indeed, we do not even know much about this in adults). One hypothesis is that children just adhere to the agreement to act together, which ensures that any event occurring in the course of the joint activity is treated under the umbrella of this agreement until both partners explicitly rescind the agreement and/or the joint goal is achieved. The finding of a previous study supports this idea by showing that a single initial agreement to play together may suffice for children to feel like they have a joint commitment and to adapt their behavior accordingly, even when players then played in parallel, that is, individually [27]. Another hypothesis is that irrespective of whether or not partners explicitly agreed to act together, a sense of less explicit solidarity, ‘we-attitude’, or group membership emerges in the course of acting together, ensuring that the partners are ready to put more effort into the activity than they would if acting alone or in parallel to each other [53]. This sense of solidarity does not have to be processed on a conscious, cognitive level but could be created by an affective rapport created on-the-fly by the partners acting together, that is, on a less explicit, affective level [54]. Because in our studies, children both agreed to play together with the other and then played the game jointly with her, we cannot tell which of these things caused children’s behavior in the collaborative condition – and of course they are not mutually exclusive. Future research should investigate this question more systematically, along with the further open question about when children (and adults) finally get fed up and stop being generous to a partner who is acting in contradiction to an established joint commitment and therefore finally withdraw from the joint activity.

In summary, these findings support and extend previous research by showing that 3-year-old children have a relatively sophisticated understanding of some of the various rights and obligations that joint commitments to act together entail. They understand that some of the consequences of joint commitments include supporting their partner through difficulties in the joint activity, taking over the other’s role when necessary, and even covering up for minor transgressions. Children thus seem to be well on their way to an adult-like understanding of the elements of joint activities.

## Supporting Information

Table S1 **Coding categories for children’s behavior in each of the five tests (Studies 1 and 2).** Children received a code for only one of all possible categories of behavior in each test.
(DOCX)

## References

[1] Brownell CA (2011) Early developments in joint action. Review of Philosophy and Psychology 2: 193–211. 23087769 10.1007/s13164-011-0056-1 PMC3474705

[2] Ratner N, Bruner J (1978) Games, social exchange and the acquisition of language. Journal of Child Language 5: 391–401. 701416 10.1017/s0305000900002063

[3] Ross HS, Lollis SP (1987) Communication within infant social games. Developmental Psychology 23: 241–248.

[4] Warneken F, Chen F, Tomasello M (2006) Cooperative activities in young children and chimpanzees. Child Development 77: 640–663. 16686793 10.1111/j.1467-8624.2006.00895.x

[5] Warneken F, Tomasello M (2007) Helping and cooperation at 14 months of age. Infancy 11: 271–294. 10.1111/j.1532-7078.2007.tb00227.x 33412734

[6] Henderson AME, Woodward AL (2011) “Let's work together”: What do infants understand about collaborative goals? Cognition 121: 12–21. 21722884 10.1016/j.cognition.2011.05.008 PMC3163231

[7] Howes C (1987) Social competence with peers in young children - Developmental sequences. Developmental Review 7: 252–272.

[8] Brownell CA, Carriger MS (1991) Collaborations among toddler peers: Individual contributions to social contexts. In: Resnick L, Levine JM, Teasley S, editors. Perspectives on socially shared cognition. Washington, DC: American Psychological Association. 365–383.

[9] Brownell CA, Ramani GB, Zerwas S (2006) Becoming a social partner with peers: Cooperation and social understanding in one- and two-year-olds. Child Development 77: 803–821. 16942491 10.1111/j.1467-8624.2006.00904.x PMC3351034

[10] Brownell CA, Carriger MS (1990) Changes in cooperation and self-other differentiation during the second year. Child Development 61: 1164–1174. 2209186

[11] Eckerman CO, Peterman K (2001) Peers and infant social/communicative development. In: Bremner G, Fogel A, editors. Blackwell handbook of infant development. Malden, MA: Blackwell. 326–350.

[12] Hunnius S, Bekkering H, Cillessen AHN (2009) The association between intention understanding and peer cooperation in toddlers. European Journal of Developmental Science 3: 368–388.

[13] Steinwender J, Warneken F, Tomasello M (2010) The development of individual and collaborative problem solving in young children. International Conference on Infant Studies in Baltimore, MD, USA.

[14] Warneken F, Gräfenhain M, Tomasello M (2012) Collaborative partner or social tool? New evidence for young children’s understanding of shared intentions in collaborative activities. Developmental Science 15: 54–61. 22251292 10.1111/j.1467-7687.2011.01107.x

[15] Bratman ME (2009) Shared agency. In: Mantzavinos C, editor. Philosophy of the social sciences: Philosophical theory and scientific practice. New York: Cambridge University Press. 41–59.

[16] Bratman ME (1992) Shared cooperative activity. Philosophical Review 101: 327–340.

[17] Searle JR (1990) Collective intentions and actions. In: Cohen PR, Morgan J, Pollack ME, editors. Intentions in communication. Cambridge, MA: MIT Press. 401–415.

[18] Pacherie E (2007) Is collective intentionality really primitive? In: Beaney M, Penco C, Vignolo M, editors. Mental processes: representing and inferring. Cambridge: Cambridge Scholars Press. 153–175.

[19] Tuomela R (1990) What are goals and joint goals? Theory and Decision 28: 1–20.

[20] Tuomela R (2005) We-intentions revisited. Philosophical Studies 125: 327–369.

[21] Tomasello M, Carpenter M, Call J, Behne T, Moll H (2005) Understanding and sharing intentions: The origins of cultural cognition. Behavioral & Brain Sciences 28: 675–735. 16262930 10.1017/S0140525X05000129

[22] Bratman ME (2009) Modest sociality and the distinctiveness of intention. Philosophical Studies 144: 149–165.

[23] Gilbert M (1990) Walking together: a paradigmatic social phenomenon. In: French PA, Uehling TE, Wettstein HK, editors. MidWest Studies in Philosophy XV, The Philosophy of the Human Sciences. Notre Dame: University of Notre Dame Press. 1–14.

[24] Gilbert M (2009) Shared intention and personal intentions. Philosophical Studies 144: 167–187.

[25] Alonso FM (2009) Shared intention, reliance, and interpersonal obligations. Ethics 119: 444–475.

[26] Tuomela R (2006) Joint intention, We-mode and I-mode. Midwest Studies in Philosophy 30: 35–58.

[27] Gräfenhain M, Behne T, Carpenter M, Tomasello M (2009) Young children's understanding of joint commitments. Developmental Psychology 45: 1430–1443. 19702403 10.1037/a0016122

[28] Hamann K, Warneken F, Tomasello M (2012) Children's developing commitments to joint goals. Child Development 83: 137–145. 22172281 10.1111/j.1467-8624.2011.01695.x

[29] Karau SJ, Williams KD (1997) The effects of group cohesiveness on social loafing and social compensation. Group Dynamics-Theory Research and Practice 1: 156–168.

[30] Williams KD, Karau SJ (1991) Social loafing and social compensation: The effects of expectations of co-worker performance. Journal of Personality and Social Psychology 61: 570–581. 1960649 10.1037//0022-3514.61.4.570

[31] DePaulo BM, Kashy DA (1998) Everyday lies in close and casual relationships. Journal of Personality and Social Psychology 74: 63–79. 9457776 10.1037//0022-3514.74.1.63

[32] Klockars CB (1984) Blue lies and police placebos - the moralities of police lying. American Behavioral Scientist 27: 529–544.

[33] Lindskold S, Han G (1986) Intent and the judgment of lies. Journal of Social Psychology 126: 129–130. 3724086 10.1080/00224545.1986.9713581

[34] Talwar V, Lee K (2002) Emergence of white-lie telling in children between 3 and 7 years of age. Merrill-Palmer Quarterly 48: 160–181.

[35] Talwar V, Murphy SM, Lee K (2007) White lie-telling in children for politeness purposes. International Journal of Behavioral Development 31: 1–11. 18997880 10.1177/0165025406073530 PMC2581483

[36] Fehr E, Bernhard H, Rockenbach B (2008) Egalitarianism in young children. Nature 454: 1079–U1022. 18756249 10.1038/nature07155

[37] Moore C (2009) Fairness in children's resource allocation depends on the recipient. Psychological Science 20: 944–948. 19515118 10.1111/j.1467-9280.2009.02378.x

[38] Warneken F, Lohse K, Melis AP, Tomasello M (2011) Young children share resources equally after collaboration. Psychological Science 22: 267–273. 21196533 10.1177/0956797610395392

[39] Hamann K, Warneken F, Greenberg J, Tomasello M (2011) Collaboration encourages equal sharing in children but not chimpanzees. Nature 476: 328–331. 21775985 10.1038/nature10278

[40] Rakoczy H (2008) Taking fiction seriously: Young children understand the normative structure of joint pretence games. Developmental Psychology 44: 1195–1201. 18605846 10.1037/0012-1649.44.4.1195

[41] Vaish A, Missana M, Tomasello M (2011) Three-year-old children intervene in third-party moral transgressions. British Journal of Developmental Psychology 29: 124–130. 21288257 10.1348/026151010X532888

[42] Lerner MJ (1974) The justice motive: “Equity” and “parity” among children. Journal of Personality and Social Psychology 29: 539–550.

[43] Blake PR, Rand DG (2010) Currency value moderates equity preference among young children. Evolution and Human Behavior 31: 210–218.

[44] Ingram GPD, Bering JM (2010) Children's tattling: The reporting of everyday norm violations in preschool settings. Child Development 81: 945–957. 20573115 10.1111/j.1467-8624.2010.01444.x

[45] Talwar V, Lee K, Bala N, Lindsay RCL (2004) Children's lie-telling to conceal a parent's transgression: Legal implications. Law and Human Behavior 28: 411–435. 15499823 10.1023/b:lahu.0000039333.51399.f6 PMC2785013

[46] Gummerum M, Keller M (2008) Moral psychology and economic game theory. European Journal of Developmental Science 2: 206–220.

[47] Kenward B, Dahl M (2011) Preschoolers distribute scarce resources according to the moral valence of recipients' previous actions. Developmental Psychology 47: 1054–1064. 21604863 10.1037/a0023869

[48] Rochat P, Dias MDG, Guo LP, Broesch T, Passos-Ferreira C, et al (2009) Fairness in distributive justice by 3-and 5-year-olds across seven cultures. Journal of Cross-Cultural Psychology 40: 416–442.

[49] denBak IM, Ross HS (1996) I'm telling! The content, context, and consequences of children's tattling on their siblings. Social Development 5: 292–309.

[50] Dunn J, Brown J, Slomkowski C, Tesla C, Youngblade L (1991) Young children's understanding of other people's feelings and beliefs: Individual differences and their antecedents. Child Development 62: 1352–1366. 1786720

[51] Hughes C, Fujisawa KK, Ensor R, Lecce S, Marfleet R (2006) Cooperation and conversations about the mind: A study of individual differences in 2-year-olds and their siblings. British Journal of Developmental Psychology 24: 53–72 (20)..

[52] Garner PW, Jones DC, Palmer DJ (1994) Social cognitive correlates of preschool children's sibling caregiving behavior. Developmental Psychology 30: 905–911.

[53] Tomasello M, Melis AP, Tennie C, Wyman E, Herrmann E (2012) Two key steps in the evolution of cooperation: The interdependence hypothesis. Current Anthropology 53: 673–692.

[54] Michael J (2011) Shared emotions and joint action. Review of Philosophy and Psychology 2: 355–373. 10.1007/s13164-011-0056-1 PMC3474705 23087769

