# Supplementary material for: Three-Year-Olds’ Understanding of the Consequences of Joint Commitments
Source: PLoS One. 2013 Sep 4;8(9):e73039. doi: 10.1371/journal.pone.0073039 (PMC3762880; doi:10.1371/journal.pone.0073039)
Supplement: Table S1 — Coding categories for children’s behavior in each of the five tests (Studies 1 and 2). Children received a code for only one of all possible categories of behavior in each test. (DOCX) [file pone.0073039.s001.docx]

# Table S1.

| **Category** | **Definition** |
| --- | --- |
|  |  |
| **Interruption test** |  |
|  | |
| *Adapting behavior to the puppet’s interruption* | |
|  |  |
| Waiting | - Child (C) starts walking, but stops at the puppet’s position (looking at her) and stays with the puppet for the majority of the time |
|  |  |
| Helping | - C helps the puppet replace her cap (e.g., gives her the cap) |
|  | - C takes one of the containers and offers it to the puppet (i.e., puts it close to the puppet or holds it out in the direction of the puppet) |
|  |  |
| *Continuing alone* | - C does not stop or only briefly stops on the way to the container or back to the experimenter (E) |
|  |  |
| *No reaction* | - C remains sitting on the floor with the experimenter |
|  |  |
| **Damage test** |  |
|  |  |
| *Helping* | - C tries to help the puppet (e.g., by picking up the board and/or collecting the pieces that had fallen out) |
|  | - C starts helping: |
|  | - before E has asked any question |
|  | - after Question 1 |
|  | - after Question 2 |
|  |  |
| *No Helping* | - C does not help the puppet |
|  |  |
| *Responses to E’s questions* | |
| Indicating | - Indicating the puppet as the source of the damage |
|  | - Verbally (e.g., “She made it fall over”, “It was her”) |
|  | - Nonverbally (pointing at the puppet, ostensive head movement in direction of the puppet) |
|  |  |
| Neutral | - Responding uninformatively (i.e., without indicating the puppet) |
|  | - Verbally (e.g., “fell over”, “It [the puzzle] just fell over”) |
|  | - Nonverbally (e.g., shrugging shoulders) |
|  |  |
| No response | - No response or response that is not clearly related to the test |
|  |  |
| **Cheating test** |  |
|  |  |
| *Responses to Questions 1 and 2* | |
| Tattling | - Tattling on the puppet (i.e., indicating the puppet) |
|  | - Verbally (e.g., “She took them [the puzzle pieces]”, “It was her”) |
|  | - Nonverbally (pointing at puppet, ostensive head movement in direction of puppet) |
|  |  |
| Neutral | - Responding uninformatively (i.e., without indicating the puppet) |
|  | - Verbally (e.g., “I don’t know”, “It [the puzzle] is just finished”) |
|  | - Nonverbally (e.g., shrugging shoulders) |
|  |  |
| No response | - No response or response that is not clearly related to the test |
|  |  |
| *Response to Question 3* |  |
| Admitting cheating | - Verbally or nonverbally (e.g., saying “yes”, nodding) |
| Denying cheating | - Verbally or nonverbally (e.g., saying “no”, shaking head) |
| No response | - No clear response to the question |
|  |  |
| **Tidying Up test** |  |
|  |  |
| *Number of blocks tidied up* |  |
| All | - C puts all the blocks in one or both containers |
| At least half | - C puts at least half (but not all) of the blocks in one or both containers |
| Less than half | - C puts less than half of the blocks in one or both containers |
|  |  |
| *Containers used to tidy up* | |
| Own | - C only puts blocks in his/her own container |
| Puppet’s | - C only puts blocks in the puppet’s container |
| Both | - C puts blocks in both containers |
|  |  |
| **Sharing test** | - Number of stickers C puts into her/his own container |
|  | - Number of stickers C puts into puppet’s container |
